# Flow diverters for intracranial aneurysm embolization with coil embolization in children with ruptured aneurysm: two cases reports

**DOI:** 10.3389/fstro.2026.1775674

**Published:** 2026-06-04

**Authors:** Quan Zhou, Shixin Jiang, Bingbing Wang, Feng Guo

**Affiliations:** 1 Shandong Second Medical University, Weifang, China; 2 Linyi People's Hospital Affiliated to Shandong Second Medical University, Linyi, China

**Keywords:** case report, flow diverter, giant intracranial aneurysm, pediatric population, subarachnoid hemorrhage

## Abstract

Pediatric intracranial aneurysms are relatively rare and exhibit distinct characteristics compared with those in adults, particularly in terms of sex distribution, incidence, anatomical location, morphology, and underlying etiology. Notably, the risk of rupture and hemorrhage is substantially higher in the pediatric population. Among children younger than five years, approximately 85% of intracranial aneurysms initially present with rupture and bleeding, whereas this proportion decreases to 45% in those older than five years, still significantly exceeding the hemorrhagic risk observed in adults. Current standard treatment modalities for intracranial aneurysms include microsurgical clipping, stent-assisted coil embolization, and flow diverter implantation. However, there are relatively few reports on the use of flow diverters in pediatric patients, and the optimal antiplatelet regimen following endovascular treatment in this population remains controversial. In this study, we report two cases of ruptured giant intracranial aneurysms in children treated with flow diverter placement combined with dense coil embolization. One patient presented with a space-occupying intracerebral hematoma and subsequently developed intracranial hypertension with cerebral herniation after the procedure, necessitating decompressive craniectomy. Different antiplatelet strategies were employed in the two cases. Both patients survived and were discharged in stable condition. At 6-month follow-up, imaging demonstrated no evidence of aneurysm recurrence, and both children had a favorable quality of life.

## Background

Pediatric intracranial aneurysms are a rare cerebrovascular disorder characterized by pathological dilation of cerebral arteries. Their pathophysiology is primarily associated with extracellular matrix degradation and remodeling, apoptosis of vascular smooth muscle cells, intimal hyperplasia, and inflammatory cell infiltration. These lesions account for approximately 0.5%−4.5% of all intracranial aneurysms (Montaser and Smith, 2021; Aguiar et al., 2022; Kommareddy et al., 2024) and differ substantially from adult aneurysms in terms of sex distribution, anatomical location, morphology, and underlying etiology (Montaser and Smith, 2021; Gemmete et al., 2013; Aguiar et al., 2022).

Pediatric aneurysms are more commonly observed in boys, with a male-to-female ratio of approximately 1.8:1 (Kommareddy et al., 2024), in contrast to the female predominance seen in adults (Goia et al., 2020). Compared with adults, children have a higher proportion of aneurysms located in the posterior circulation, often with more complex morphologies. Dissecting and pseudoaneurysms are more frequently encountered, whereas typical saccular aneurysms are less common (Agid et al., 2007; Allison et al., 1998). In addition, large and giant aneurysms constitute a significant proportion of pediatric cases (Scoville et al., 2021). Despite these differences, long-term outcomes in children are generally more favorable than in adults (Clarke et al., 2022).

In children younger than five years, up to 85% of intracranial aneurysms initially present with rupture and hemorrhage, with this proportion decreasing with age. The overall mortality rate ranges from 10% to 23% (Heredia-Gutiérrez and Carbarín-Carbarín, 2021). Approximately 27% of pediatric patients with subarachnoid hemorrhage develop concomitant intracerebral hematoma (Aguiar et al., 2022; Agid et al., 2007; Allison et al., 1998; Pasqualin et al., 1986; Beez et al., 2016; Xu et al., 2021), which is associated with younger age and distal arterial location (Scoville et al., 2021). Most survivors experience varying degrees of neurological disability, significantly impacting quality of life.

Current treatment strategies primarily include endovascular embolization and microsurgical clipping. However, the use of flow diverters in pediatric patients remains off-label and is infrequently reported. The two cases presented in this study both met the criteria for large or giant aneurysms, with one complicated by intracerebral hematoma. These cases provide additional evidence supporting the therapeutic potential of flow diverters in pediatric intracranial aneurysms. Furthermore, by comparing two different antiplatelet regimens, this study explores the safety and feasibility of antiplatelet therapy following endovascular treatment in the pediatric population.

## Case Report

### Case 1

A 2-year-old boy was admitted with a 3-hour history of irritability and seizures. On physical examination, the patient was comatose with a Glasgow Coma Scale (GCS) score of 3. Both pupils were equal and round, measuring approximately 3.5 mm in diameter, with preserved light reflexes. Neck stiffness was present. Increased muscle tone was noted in both lower extremities, with hyperactive knee reflexes and a positive Babinski sign. The patient was classified as Hunt–Hess grade IV and Fisher grade III.

**Figure 1:**
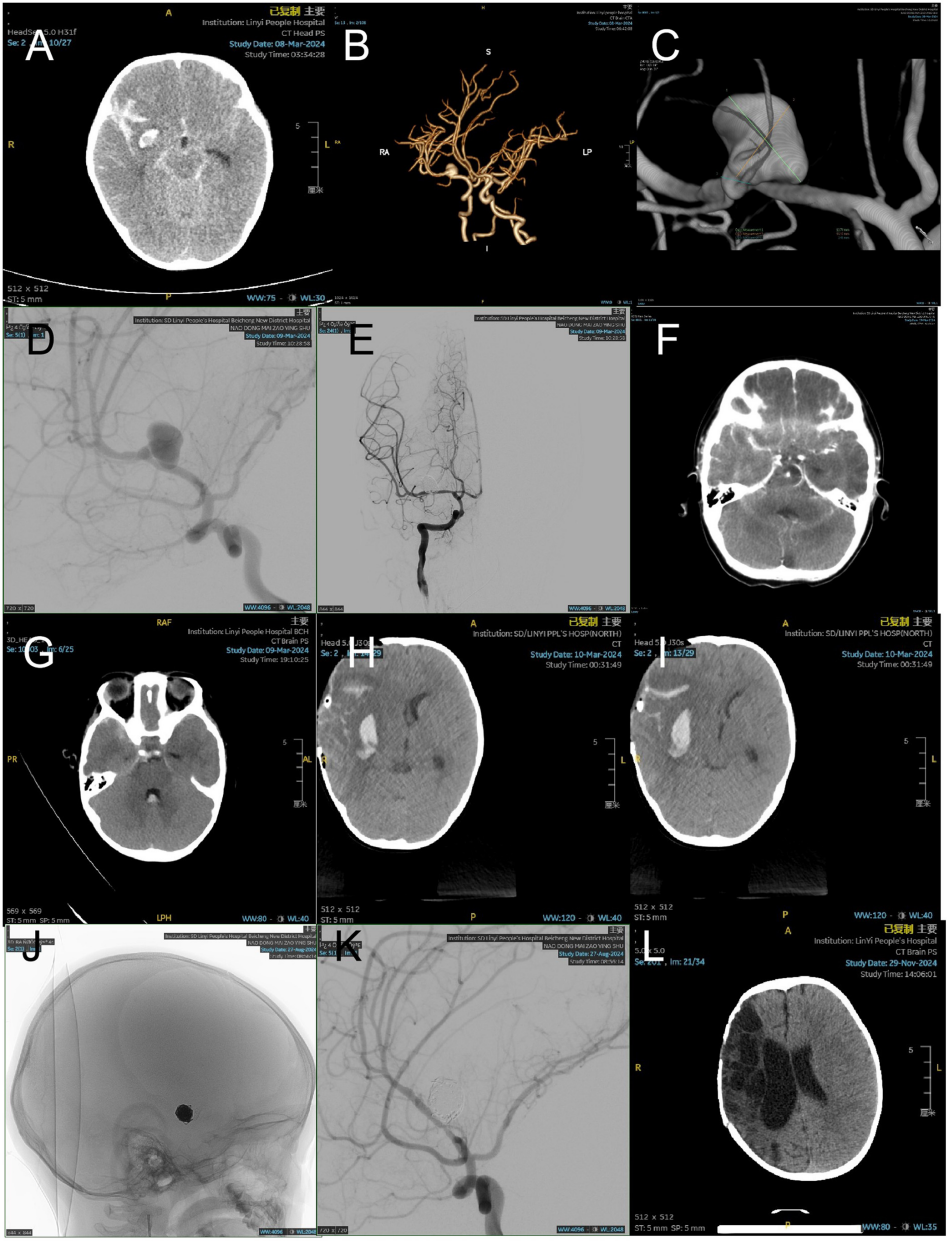
Imaging of case 1. **(A, B)** Initial cranial CT angiography (CTA) on admission demonstrating a right middle cerebral artery (MCA) aneurysm with subarachnoid hemorrhage. **(C, D)** Cerebral digital subtraction angiography (DSA) showing an aneurysm located in the M1 segment of the right MCA. **(E)** Immediate post-embolization angiography demonstrating dense packing of the aneurysm. **(F)** Immediate post-procedural CT following intracranial aneurysm embolization. **(G)** CT scan obtained after sudden onset of pupillary dilation. **(H, I)** CT scans following decompressive craniectomy. **(J, K)** Follow-up DSA demonstrating complete obliteration of the aneurysm, with mild proximal stenosis of the parent artery. **(L)** CT scan after cranioplasty.

Initial head CT revealed subarachnoid hemorrhage, and subsequent CT angiography (CTA) demonstrated a giant aneurysm at the bifurcation of the right middle cerebral artery, with features suggestive of a possible dissecting aneurysm (Figures 1A, B). Endovascular treatment with a flow diverter combined with coil embolization was therefore planned.

Intraoperative digital subtraction angiography (DSA) confirmed an aneurysm located at the M1 segment of the right middle cerebral artery, with a neck measuring 3.48 mm and an overall size of approximately 12.78 × 10.10 mm. The aneurysm exhibited a highly irregular morphology, and lenticulostriate arteries were observed originating from the aneurysm neck (Figures 1C, D).

During the procedure, seven coils were deployed via an Echelon-10 microcatheter to achieve embolization of the aneurysm sac. Subsequently, a Tubridge flow diverter (2.5 mm × 15 mm) was delivered through a Fastrack microcatheter and successfully deployed across the aneurysm neck. Post-deployment angiography demonstrated complete expansion of the stent, good patency of the parent artery, and dense embolization of the aneurysm (Figure 1E). Postoperative CT scan showed no increase in subarachnoid hemorrhage (Figure 1F). The procedure was completed at 12:00 on the same day.

Immediately after stent deployment, 1.5 mL of tirofiban hydrochloride solution (5 mg/100 mL) was administered intravenously, followed by continuous infusion at the 1.5ml/h. Postoperatively, the patient was transferred to the intensive care unit for management including dehydration therapy, intracranial pressure control, life support, antiplatelet therapy, and anti-infective treatment.

On the night following surgery, the patient developed acute right-sided pupillary dilation. Emergency head CT revealed worsening cerebral edema, and decompressive craniectomy was performed (Figure 1G). Post-craniotomy bone flap removal CT scan: exacerbation of cerebral edema (Figures 1H, I). Supportive care was continued postoperatively. At 08:00 on postoperative day 1, 75 mg of aspirin was administered via nasogastric tube, and tirofiban infusion was discontinued at 12:00. Thereafter, aspirin was maintained at 25 mg once daily via nasogastric or oral administration.

The patient's condition gradually improved, and he was transferred out of the intensive care unit on postoperative day 8. Due to left-sided hemiparesis, he was transferred to the pediatric rehabilitation department on postoperative day 18 for further treatment. The total hospital stay was 19 days.

At 6-month follow-up, the patient was readmitted for digital subtraction angiography and cranioplasty. On admission, he was conscious, in good general condition, and had clear speech. Muscle strength in the left upper limb was graded as 3/5, while muscle strength and tone in the remaining limbs were within normal limits. Follow-up angiography demonstrated complete occlusion of the previously treated aneurysm, with mild stenosis of the proximal parent artery (Figures 1J, K). Cranioplasty was successfully performed (Figure 1L), and the patient was discharged on postoperative day 6 with ongoing rehabilitation.

### Case 2

A 10-year-old girl was admitted with a 2-day history of headache, vomiting, and progressive visual impairment. On examination, she was conscious but in poor general condition, with mildly impaired speech fluency. Both pupils were round and equal, measuring approximately 2 mm in diameter, and extraocular movements were grossly intact. Muscle strength and tone in all extremities were normal, no pathological reflexes were elicited, and Kernig's sign was equivocal (±). The patient was classified as Hunt–Hess grade I, Fisher grade I, with a Glasgow Coma Scale (GCS) score of 15.

**Figure 2:**
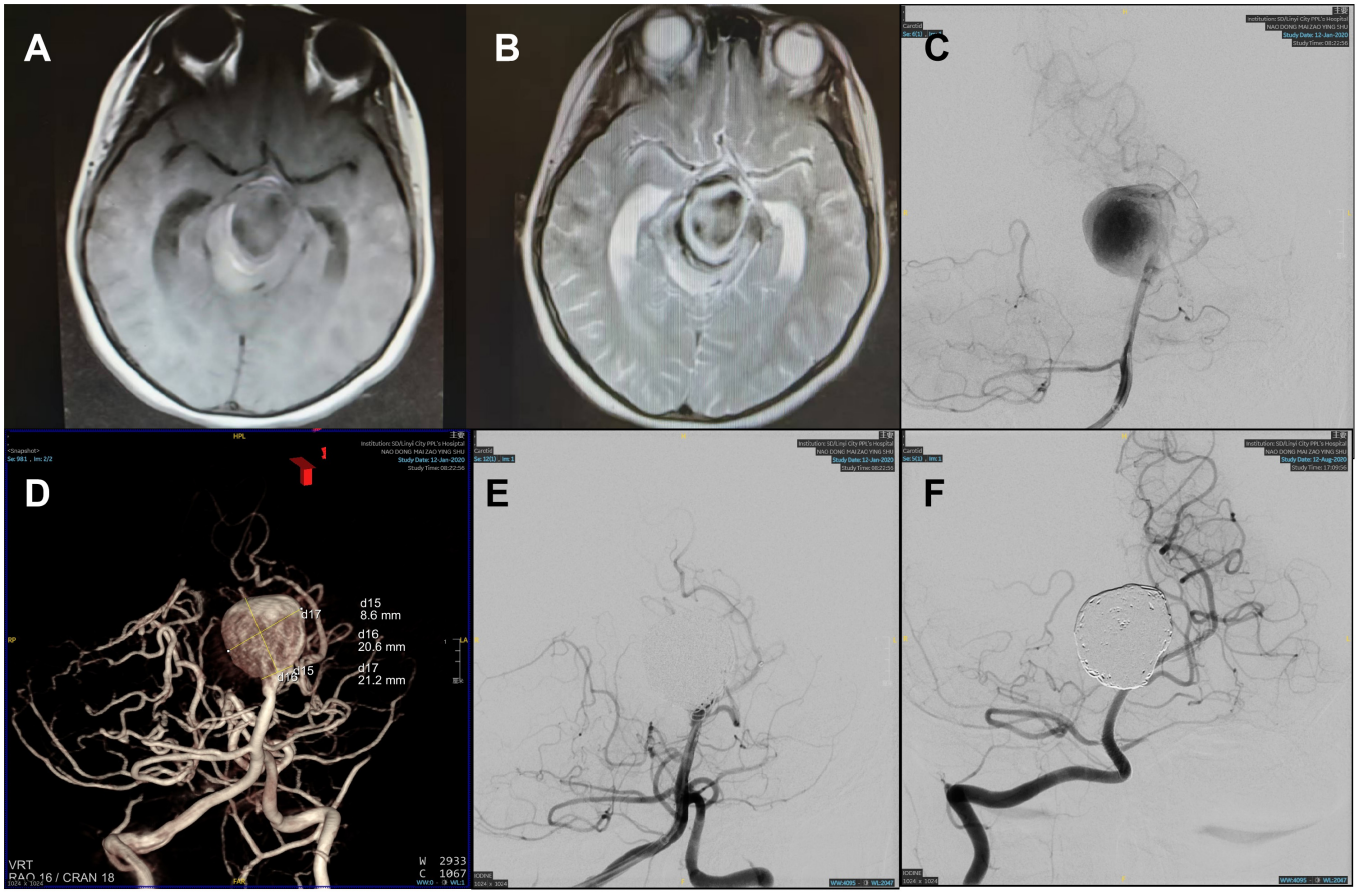
Imaging of case 2. **(A, B)** MRA demonstrating a basilar apex aneurysm with associated subarachnoid hemorrhage. **(C, D)** Preoperative DSA showing a giant basilar apex aneurysm. **(E)** Immediate post-embolization DSA demonstrating complete aneurysm occlusion with good visualization of the parent artery. **(F)** Six-month follow-up DSA showing persistent aneurysm occlusion with a patent parent artery and no evidence of stenosis.

Magnetic resonance angiography (MRA) performed at an outside institution demonstrated a basilar apex aneurysm associated with subarachnoid hemorrhage (Figures 2A, B). Following admission, digital subtraction angiography (DSA) confirmed a giant aneurysm at the basilar artery apex, with a wide neck measuring 8.6 mm and an overall size of approximately 25.2 × 20.2 mm, projecting posterosuperiorly. The right posterior cerebral artery was not visualized (Figures 2C, D).

After comprehensive evaluation, endovascular treatment with a flow diverter combined with coil embolization was performed. A loading dose of dual antiplatelet therapy (aspirin 300 mg and clopidogrel 300 mg) was administered orally 2 hours prior to the procedure. Intraoperatively, a Pipeline Embolization Device (PED; 3.75 mm × 35 mm) was deployed, and 21 coils were sequentially delivered via an Echelon-10 microcatheter to achieve dense embolization of the aneurysm sac. Post-procedural angiography demonstrated satisfactory occlusion of the aneurysm and good patency of the parent artery (Figure 2E).

The patient was transferred to the intensive care unit postoperatively, where she received management including dehydration therapy, intracranial pressure control, life support, antiplatelet therapy, and anti-infective treatment. From postoperative day 2 onward, dual antiplatelet therapy (aspirin 50 mg plus clopidogrel 50 mg daily) was initiated.

On postoperative day 7, the patient's vital signs were stable, and she was transferred to the general ward. She was discharged on postoperative day 18 and continued rehabilitation at a local hospital, with a total hospital stay of 23 days.

At 6-month follow-up, the patient was alert, in good general condition, with fluent speech and no significant abnormalities in muscle strength or tone. Follow-up DSA demonstrated complete obliteration of the basilar apex aneurysm, with preserved patency of the parent artery and no evidence of stenosis (Figure 2F).

## Discussion and Conclusion

There is currently no established consensus for the treatment of pediatric intracranial aneurysms, with most previous cases being managed through craniotomy with clipping or coil embolization (Montaser and Smith, 2021). However, due to the complex nature of pediatric aneurysms, recurrence rates remain high. In recent years, the flow diverter, a novel endovascular device, has been approved for use in adults but remains off-label in pediatric cases. The flow diverter promotes thrombosis and occlusion within the aneurysm through a blood flow diversion mechanism, making it particularly suitable for treating complex aneurysm cases (Csippa et al., 2020). For example, in a case of a giant cavernous segment carotid artery aneurysm in a child, successful aneurysm occlusion was achieved using a flow diverter, and follow-up angiography showed favorable results (Kumaria et al., 2021). Similarly, another study reported two pediatric patients treated with the Pipeline Embolization Device (PED), both of whom achieved complete aneurysm occlusion and excellent clinical outcomes, highlighting the potential of flow diverters in cases where traditional treatments were ineffective (Eraky et al., 2024). These cases suggest that flow diverters may offer an effective alternative for managing complex pediatric aneurysms (Sousa et al., 2024).

Most previous reports focus on unruptured aneurysms, with one of the largest series reporting 50 intracranial aneurysms, only 5 of which were treated acutely after rupture (Cherian et al., 2020). In contrast, the two cases presented in this study both involved ruptured aneurysms, with one patient also developing an intracerebral hematoma. To minimize the risk of aneurysm recurrence, both patients underwent coil embolization combined with flow diverter placement, achieving satisfactory results on follow-up imaging.

In terms of safety, a systematic review analyzing 37 pediatric cases from 2007 to 2019 found a complication rate of 21.6%, including death, residual aneurysms, and parent artery occlusion (Scoville et al., 2021). Regarding ruptured intracranial aneurysms, there is ongoing debate on whether to use coils in combination with flow diverters during treatment. Some studies have suggested that the use of coils with a flow diverter reduces the risk of surgical and delayed complications without increasing the occlusion rate of the aneurysm (Sein et al., 2025). For devices such as the Tubridge and PED, endothelial cell proliferation and aneurysm thrombosis generally occur within about 6 months. Thus, our center employs dense coil embolization in conjunction with the flow diverter to prevent re-bleeding and reduce the recurrence rate.

Another consideration is the lack of established guidelines for antiplatelet therapy in children. Different studies report varying doses of antiplatelet medications (Cobb et al., 2017; Samaniego et al., 2019). One study on pediatric cardiac patients suggested that a dose of 0.2 mg/kg of clopidogrel is sufficient for therapeutic effects. However, there is currently no research addressing antiplatelet therapy specifically for intracranial stenting, especially with flow diverters. One study indicated that tirofiban and dual antiplatelet therapy (DAPT) are safe, with no significant effects on platelet count or hemoglobin levels, and no increased incidence of symptomatic bleeding or thromboembolic complications (Samaniego et al., 2019).

In Case 1, since the embolization procedure was performed immediately after cerebral angiography, our center administered 1.5 mL of tirofiban hydrochloride solution (5 mg/100 mL) intravenously during the procedure, followed by continuous infusion at 1.5 mL/h. In Case 2, embolization was delayed by two days after angiography, so the patient received a triple loading dose of dual antiplatelet therapy (aspirin 300 mg + clopidogrel 300 mg) before the procedure, followed by standard dual antiplatelet therapy postoperatively (Texakalidis et al., 2017). We believe that although oral antiplatelet therapy before surgery is safe, in cases where surgical conversion to craniotomy is needed due to difficulties with the endovascular approach, oral dual antiplatelet therapy may pose a risk. Thus, we recommend intraoperative anticoagulation, followed by double or triple doses of antiplatelets after the procedure, with cessation of systemic anticoagulation after 12 hours. Subsequent antiplatelet therapy is administered at the standard dose after 48 hours. In our cases, antiplatelet therapy was calculated based on adult dosages, adjusted for body weight, and no bleeding or ischemic complications were observed.

### Conclusion

the cases presented here, in conjunction with previous reports, demonstrate that flow diverters show promising safety and efficacy for treating pediatric intracranial aneurysms. However, due to the rarity of pediatric aneurysms, larger-scale, multicenter trials are needed to compare the outcomes of flow diverters with other treatment options (Eraky et al., 2024). Additionally, the use of tirofiban during the procedure or a triple loading dose of dual antiplatelet therapy before surgery appears to be both safe and effective.

## Data Availability

The datasets presented in this article are not readily available because of ethical and privacy restrictions. Requests to access the datasets should be directed to the corresponding author.
